# Accounting for intervention complexity in rcts in surgery: new approaches for intervention definition and methods for monitoring fidelity

**DOI:** 10.1186/1745-6215-14-S1-O86

**Published:** 2013-11-29

**Authors:** Natalie Blencowe, Alex Boddy, Alexander Harris, Tom Hanna, Penny Whiting, Jonathan Cook, Jane Blazeby

**Affiliations:** 1 University of Bristol, Bristol, UK; 2 Imperial College London, London, UK; 3 University Hospitals Bristol NHS Foundation Trust, Bristol, UK; 4 Royal Cornwall Hospitals Trust NHS, Truro, UK; 5 University of Aberdeen, Aberdeen, UK

## 

The recognition that surgical interventions are complex has major implications for the design of RCTs, including the need for methods to describe interventions in study protocols to allow delivery and fidelity to be accurately assessed. We report approaches to defining and describing surgical interventions and assessing intervention fidelity.

Some 81 RCTs evaluating 135 surgical interventions, published between 2010 and 2011, were systematically identified. A subset of reports were scrutinised and iterative discussions developed a classification framework for intervention definition and fidelity. Two researchers independently read and re-read articles, discussed with the research team, and re-worked the classification to inform the framework which was reapplied to all papers.

The whole surgical intervention, component parts and individual steps were classified. Whole interventions were categorised into four groups: i) resection, ii) reconstruction, iii) resection and reconstruction, and iv) exploration. Components of interventions included i) incision, ii) dissection, iii) haemostasis, and iv) closure. Individual steps within each component included categories such as length of incision or extent of dissection. Descriptions of whole interventions, component parts and individual steps were each classified as mandatory, prohibited or optional. Mandatory elements included those delivered flexibly, within limits, or exactly. Intervention fidelity was similarly categorised as relating to the whole intervention, component parts and individual steps.

Descriptions and categorisation of surgical interventions is feasible and the precise level of detail required will depend upon trial design and the nature of the research question. Further work to explore the application of this system to new trials is now required.

